# Identification of master regulator genes controlling pathogenic CD4^+^ T cell fate in inflammatory bowel disease through transcriptional network analysis

**DOI:** 10.1038/s41598-024-61158-4

**Published:** 2024-05-08

**Authors:** José M. Jiménez, J. Sebastián Contreras-Riquelme, Pía M. Vidal, Carolina Prado, Macarena Bastías, Claudio Meneses, Alberto J. M. Martín, Tomás Perez-Acle, Rodrigo Pacheco

**Affiliations:** 1 Centro Científico y Tecnológico de Excelencia Ciencia & Vida, Fundación Ciencia & Vida, Avenida Del Valle Norte #725, 8580702 Huechuraba, Santiago Chile; 2 Centro de Biotecnología Vegetal, Universidad Andrés Bello, Santiago, Chile; 3 Biomedical Science Research Laboratory, Neuroimmunology and Regeneration of the Central Nervous System Unit, Basic Sciences Department, Faculty of Medicine, Universidad Católica de la Santísima Concepción, Concepción, Chile; 4 Facultad de Medicina y Ciencia, Universidad San Sebastián, 7510156 Providencia, Santiago Chile; 5 Facultad de Ciencias Biológicas, Pontificia Universidad Católica de Chile, Santiago, Chile; 6 Facultad de Agronomía y Sistemas Naturales, Pontificia Universidad Católica de Chile, Santiago, Chile; 7 Escuela de Ingeniería, Facultad de Ingeniería Arquitectura y Diseño, Universidad San Sebastián, Santiago, Chile

**Keywords:** Inflammatory bowel diseases, CD4^+^ T cells, Gut inflammation, Master regulator genes, *Lef1*, *Mybl2*, Computational biology and bioinformatics, Immunology, Gastroenterology

## Abstract

Inflammatory bowel diseases (IBD) are a group of chronic inflammatory conditions of the gastrointestinal tract associated with multiple pathogenic factors, including dysregulation of the immune response. Effector CD4^+^ T cells and regulatory CD4^+^ T cells (Treg) are central players in maintaining the balance between tolerance and inflammation. Interestingly, genetic modifications in these cells have been implicated in regulating the commitment of specific phenotypes and immune functions. However, the transcriptional program controlling the pathogenic behavior of T helper cells in IBD progression is still unknown. In this study, we aimed to find master transcription regulators controlling the pathogenic behavior of effector CD4^+^ T cells upon gut inflammation. To achieve this goal, we used an animal model of IBD induced by the transfer of naïve CD4^+^ T cells into recombination-activating gene 1 (*Rag1*) deficient mice, which are devoid of lymphocytes. As a control, a group of *Rag1*^*−/−*^ mice received the transfer of the whole CD4^+^ T cells population, which includes both effector T cells and Treg. When gut inflammation progressed, we isolated CD4^+^ T cells from the colonic lamina propria and spleen tissue, and performed bulk RNA-seq. We identified differentially up- and down-regulated genes by comparing samples from both experimental groups. We found 532 differentially expressed genes (DEGs) in the colon and 30 DEGs in the spleen, mostly related to Th1 response, leukocyte migration, and response to cytokines in lamina propria T-cells. We integrated these data into Gene Regulatory Networks to identify Master Regulators, identifying four up-regulated master gene regulators *(Lef1, Dnmt1, Mybl2,* and *Jup*) and only one down-regulated master regulator (*Foxo3*). The altered expression of master regulators observed in the transcriptomic analysis was confirmed by qRT-PCR analysis and found an up-regulation of *Lef1* and *Mybl2*, but without differences on *Dnmt1, Jup,* and *Foxo3*. These two master regulators have been involved in T cells function and cell cycle progression, respectively. We identified two master regulator genes associated with the pathogenic behavior of effector CD4^+^ T cells in an animal model of IBD. These findings provide two new potential molecular targets for treating IBD.

## Introduction

Inflammatory bowel diseases (IBD), including mainly Crohn’s disease (CD) and ulcerative colitis (UC), are a group of chronic inflammatory conditions of the gastrointestinal tract associated with multiple pathogenic factors, including dysregulation of immune response^1,2^. Recent epidemiological studies have indicated that over 6.8 million individuals are affected by IBD worldwide, emerging as a significant public health concern around the globe^3^.

The gastrointestinal tract harbors interactions between beneficial microbes and potential pathogens. In IBD, this equilibrium is disturbed, causing a prolonged immune attack on the intestinal microbiota. Genetic susceptibilities, combined with environmental triggers, might result in the activation of both the innate and adaptive immune system, perpetuating mucosal inflammation and tissue damage^4^. In this context, effector CD4^+^ T cells (Teff, which display an inflammatory phenotype) and regulatory CD4^+^ T cells (Treg, which display a suppressive phenotype) constitute central players in maintaining the equilibrium between tolerance and inflammation^5^. Teff, particularly Th1 and Th17 subsets, play pivotal roles in mediating this inflammatory cascade, leading to pathological conditions^6^.

Interestingly, genetic modifications on these cells have been implicated in regulating the commitment of specific phenotypes and immune functions^7^. However, the transcriptional program controlling the pathogenic behavior of T helper cells in IBD progression is still unknown^8,9^. Thus, deciphering how gene regulatory networks dictate the effector or suppressive function of T lymphocytes in IBD constitutes a novel way to find key check-points controlling IBD pathogenesis. Indeed, master regulator genes have gained significant attention because of their crucial function in directing gene expression in both homeostatic and pathologic conditions, given their ability to control the simultaneous expression of multiple genes^10^.

Here, we aimed to identify the key master regulators that govern the pathological phenotype of CD4^+^ T cells in intestinal inflammation. To this end, we carried out transcriptomic studies on CD4^+^ T cells extracted from both colonic and splenic tissues of mice undergoing inflammatory colitis or upon healthy conditions. Subsequently, we used a systems biology strategy to develop a master gene network to discern the transcription factors orchestrating the inflammatory condition. Our findings revealed a marked disparity in RNA expression between CD4^+^ T cells obtained from the colon compared to their splenic counterparts, with a significant upregulation of proinflammatory and leukocyte migration pathways in colonic lamina propria tissue. Notably, our transcriptomic network analysis identified five master regulators, with Lef1 and Mybl2 emerging as promising candidates for further exploration in IBD research.

## Materials and methods

### Animals

Ten to twelve-week-old female mice were used in all experiments. *Foxp3*^*GFP*^ reporter mice obtained from the Jackson Laboratories^11^ were used as donors. Recombination activating gene 1 deficient (*Rag1*^*−/−*^) mice obtained from the Jackson Laboratories^12^ were used as recipients. All mouse strains were in the C57Bl/6 genetic background. This study is reported in accordance with ARRIVE guidelines.

### Isolation of colonic and spleen lymphocytes

The isolation of lamina propria lymphocytes from the colon was performed as previously described with minor modifications^13^. Briefly, colons were cut open longitudinally and washed with cool HBSS containing 10 mM HEPES to remove feces and debris. Then, colons were incubated in HBSS containing 10 mM HEPES, 5 mM EDTA, 1 mM DTT and 5% FBS for 20 min at 37 °C, vortexed gently during the final 20 s, and the supernatant was collected. This procedure was repeated twice. The colonic tissues were placed on gentleMACS tubes and digested in HBSS containing 10 mM HEPES, 0.5 mg/ mL Collagenase D, 0.5 mg/mL DNase I grade II, 3 mg mL liberase (Roche), and 5% FBS for 30 min at 37 °C on a shaking platform. After running gentleMACS intestine dissociator program, the digested tissues containing colonic lamina propria lymphocytes were passed through a cell strainer. Leukocytes were further enriched by Percoll gradient centrifugation (44%/67%). On the other hand, the isolation of lymphocytes from spleen was performed by CD4^+^ T cell isolation kit following the manufacturer instructions (Miltenyi, Biotech, CA, USA). Once isolated, both lymphocytes types were stained and subjected to cell sorting using a FACSAria II (BD, NJ, USA).

### Antibodies and cell sorting

Fluorochrome-conjugated monoclonal antibodies (mAb) specific to mouse CD3 (clone 145-2C11), CD4 (clone GK-1.5), TCRβ (clone B183983), CD44 (clone IM7), CD62L (clone MEL-14) were purchased from Biolegend. Cells were stained over 15 min at 4 °C and then washed with PBS-FBS 5% before sorting as described before^14^. The naïve T cells population isolated from *Foxp3*^*GFP*^ mice was defined as CD3^+^CD4^+^CD62L^+^CD44^-^GFP^-^, whereas total CD4^+^ T cells were defined as CD3^+^CD4^+^. These T cell populations were sorted using a FACSAria II equipment (BD).

### T cell transfer induced colitis

Chronic inflammatory colitis was induced as described previously^14,15^. Briefly, *Rag1*^*−/−*^ recipient mice received the i.v. transfer of naïve CD4^+^ T cells or total CD4^+^ T cells (5 × 10^5^ cells per mouse) as control (Fig. S1), and body weight of each animal was recorded weekly. Four animals per tissue were analyzed for each experimental group. After 10 to 12 weeks, mice were euthanized to obtain lymphocytes from spleen and colonic lamina propria for subsequent analysis. The expression of phenotypic markers and the frequency of transferred T cells were assessed by flow cytometry and purified by cell-sorting (Fig. S1). Due to the sample exclusion process based on the RNA quality threshold, some animals were used to collect T cells from both the spleen and colonic lamina propria, whereas other animals were used to collect T cells only from the spleen or colonic lamina propria.

### RNA isolation and sequencing

Total RNA was isolated from CD4^+^ T cells using the EZNA total RNA kit I (Omega BioTek, Norcross, GA, USA) following the manufacturer’s instructions. Genomic DNA digestion was then performed using the Ambion TURBO DNA-free kit as recommended (Life Technologies, Carlsbad, CA, USA). RNA quantification and quality control were conducted in an Agilent Fragment Analyzer, using the Agilent HS RNA 15nt kit (Santa Clara, CA, USA), with an RQN > 7.0 established as the minimal quality threshold for library preparation. Subsequently, complementary DNA and the final RNA sequencing library were generated from 100 ng of total RNA using the TruSeq RNA library prep kit v2 (Illumina, San Diego, CA, USA). The RNA sequencing libraries were conducted using the Illumina NovaSeq 6000 with a sequencing depth of 20-25M PE100 paired end reads per sample.

### Transcriptomic analysis

Raw data quality was checked with FastQC (http://www.bioinformatics.babraham.ac.uk/projects/fastqc/). Then, adapters and low-quality bases (Phred score < 30) were trimmed using Trimmomatic (v. 0.39)^16^. Filtered reads were mapped to the mouse genome (GRCm38/mm10 version) using STAR (v. 2.7.10b)^17^ and gene counts were computed according to HTSeq-count (v. 0.11.1)^18^. The batch effect was adjusted with Combat-seq of the sva R package (v. 3.48.0)^19^ and finally, the differential expression of the libraries was analyzed with the DESeq2 (v. 1.40.2) package^20^, using an adjusted Pvalue cutoff of 0.05.

For those differentially expressed genes, both in spleen and colon, an enrichment analysis of biological processes was performed using the enrichGO function from the R library clusterProfiler (v. 4.8.3)^21,22^. A p-value cutoff of 0.05, Benjamini–Hochberg adjustment method, and the annotation database available in the R library org.Mm.eg.db (v. 3.17.0) were employed (https://bioconductor.org/packages/release/data/annotation/html/org.Mm.eg.db.html).

### Development of gene regulatory networks and inference of master regulators

To model the regulatory interactions in CD4^+^ T-cells isolated from control and IBD mice, we considered as reference network the union of high confidence interactions deposited in the TRRUST, RegNetwork and DoRothEA^23–25^. To contextualize this reference Gene Regulatory Network (GRN), we kept the most probable regulations by removing outgoing edges of non-expressed transcriptional factors, using as the cutoff of expression a value of 10 raw counts as previously described^26,27^.

We searched contextualized GRNs for the master regulators of each condition studied. This process was carried out selecting the first and second upstream neighbors of differentially expressed genes in the GRNs, and then looking for transcription factors with the highest edge density following the master regulator definition described by Davis and Rebay^28^. To perform this task, we delete nodes with lower out-degree iteratively, until removing a node results in an unconnected network. For the remaining transcription factors, we observed documented properties of master regulators in terms of directing and being directed by other candidate to be a master regulator, while also examining physical interactions among the protein products^28^ deposited in the STRING database^29^. The contextualized and master regulators network for colon and spleen can be found in Supplementary information. These files include Cytoscape sessions (.cys files) for visualization using Cytoscape (https://cytoscape.org/index.html), as well as graphml format files for visualization using online tools such as Gephi (https://gephi.org/gephi-lite/). Further information can be found in the respective readme files.

### Real‐time quantitative RT-PCR

qRT-PCR quantifications were performed as described before^13^. Total RNA was treated with DNase using the Ambion TURBO DNA-free kit (Life Technologies) and then used to synthesize cDNA catalyzed by the M-MLV reverse transcriptase (Life Technologies). Quantitative gene expression analysis was performed using Brilliant II SYBR Green QPCR Master Mix (Agilent). Expression of target genes was normalized to levels of *Gapdh* transcripts and compared to control values using delta delta CT method^30^. RNA samples used for qRT-PCR came from a small countersample of the RNA obtained from CD4^+^ T cells isolated from each tissue used for RNAseq. Of note, all four RNA countersamples from the control group were available, but only three RNA countersamples were available from the colitis group for qRT-PCR analysis. The primers used are summarized in the next table:

**Table:** 

Gene	Primer Sequence (5′ ➔ 3′)
*Lef1*	Forward: GCCACCGATGAGATGATCCC Reverse: TTGATGTCGGCTAAGTCGCC
*Mybl2*	Forward: TCTGGATGAGTTACACTACCAGG Reverse: GTGCGGTTAGGAAAGTGACTG
*Dnmt1*	Forward: ATCCTGTGAAAGAGAACCCTGT Reverse: CCGATGCGATAGGGCTCTG
*Jup*	Forward: TGGCAACAGACATACACCTACG Reverse: GGTGGTAGTCTTCTTGAGTGTG
*Foxo3*	Forward: CTGGGGGAACCTGTCCTATG Reverse: TCATTCTGAACGCGCATGAAG
*Gapdh*	Forward: TCCGTGTTCCTACCCCCAATG Reverse: GAGTGGGAGTTGCTGTTGAAG

### Statistical analysis

The results of mice body weight and qPCR relative expression are expressed as the mean ± SEM. Comparisons between two groups were performed using unpaired Student’s *t*-test. Statistical significance was defined at *p* < 0.05. Analyses were performed using GraphPad Prism 9 software (CA, USA), available at https://www.graphpad.com.

### Ethics approval and consent to participate

All procedures and housing were compliant with the recommendations in the 8th edition of the Guide for the Care and Use of Laboratory Animals and with the United States Public Health Service Policy. The protocol was approved by the IACUC of Fundación Ciencia & Vida (Permit Number: P0016/2017).

## Results

### Transcriptomic analysis of effector CD4^+^ T cells upon intestinal inflammation reveals pronounced transcriptional changes in colonic lymphocytes compared to splenic lymphocytes

**Figure 1:**
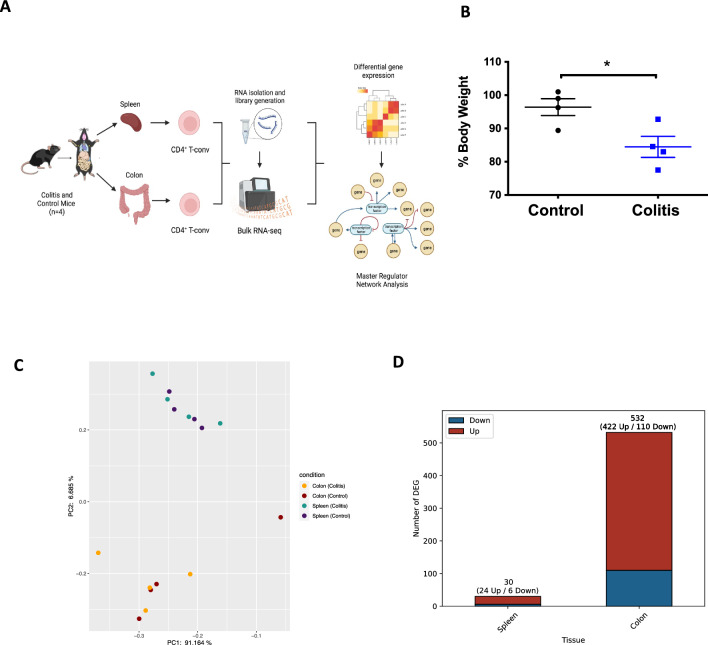
Bulk RNAseq analysis of splenic and colonic CD4^+^ T cells from colitis and control mice. (**A**) Illustration of the experimental strategy conducted to determine the differential gene expression and to built the master regulator network. (**B**) Quantification of body weight loss in experimental mice. Mean ∓ SEM are indicated. *, *p* < 0.05 by Student's t-test. (**C**) Principal Component Analysis (PCA) of RNAseq-based expression data from splenic and colonic samples from mice undergoing colitis or from healthy controls. (**D**) Quantification of differentially expressed genes (DEGs) found in splenic and colonic CD4^+^ T cells from mice undergoing colitis respect to the control conditions (n = 4). DEG upregulated are represented in red, whereas DEG downregulated are represented in blue.

To characterize the transcriptional profile of CD4^+^ T cells under gut inflammation conditions, we isolate conventional CD4^+^ T cells (CD4^+^TCRb^+^GFP^-^) from colonic lamina propria. Despite mesenteric lymph nodes, which drain the colonic tissue, might be considered a proper control to compare what is happening in the lamina propria^31^, we chose splenic CD4^+^ T cells as a control since our previous studies have shown a similar profile between T cells isolated from colonic lamina propria and mesenteric lymph nodes, but different from those isolated from the spleen upon gut inflammation^14,32^. Then, we performed bulk RNA sequencing on these samples, and then compared the inflamed condition with the control. Next, we analyzed context specific gene regulatory networks to identify master regulators (Fig. 1A). The loss of body weight was confirmed in mice undergoing colitis (Fig. 1B). In our exploration of the transcriptomic landscape across collected samples, Principal Component Analysis (PCA) was employed to discern overarching patterns of gene expression variance. The PCA plot revealed a clear separation between sample sources (colon and spleen) rather than disease conditions (Fig. 1C), suggesting a tissue-specific expression pattern. To study whether the profile expression of colonic and spleen CD4^+^ T cells is altered in inflammation, we applied the DESEq-2 algorithm to define differentially expressed genes (DEG) in both conditions. We found that colonic lamina propria CD4^+^ T cells from colitis mice showed 532 DEG compared with healthy controls. Whereas, only 30 DEG were found in spleen CD4^+^ T cells from colitis mice compared to the respective normal condition (Fig. 1D). In Table S1, we show the list of 422 genes up-regulated and 110 genes down-regulated in CD4^+^ T cells from inflamed colonic lamina propria, and in supplemmentary Table S2 we provide a shorter list of 24 genes up-regulated and 6 genes down-regulated in CD4^+^ T cells from spleen.

### Gene expression in colonic CD4^+^ T cells during colitis exhibit Th1-like phenotype

**Figure 2:**
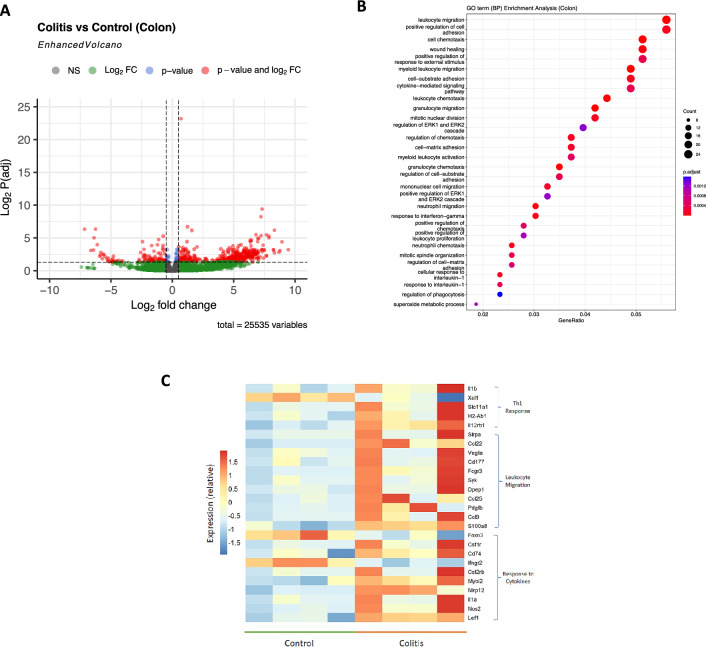
RNAseq analysis of colonic CD4^+^ T cells reveals a Th1-like phenotype under inflammatory condition. (**A**) Volcano plot of differentially expressed genes in CD4^+^ T cells from colitis matched to control, showing log_2_fold change vs -log_2_ P (adj), using Deseq-2 algorithm. In red circles are shown genes differentially expressed. Dashed lines indicate the *x*-axis threshold of |log_2_[fold change]| > 2 and the *y*-axis threshold of log_2_P(adj) < 0.05. (**B**) Gene ontology enrichment analysis of gene cluster altered in colonic CD4^+^ T cells from inflamed tissue. (**C**) Heat-map of a selected gene set of colonic CD4^+^ T cell, showing main genes related to Th1 response, leukocyte migration and response to cytokines (n = 4).

**Figure 3:**
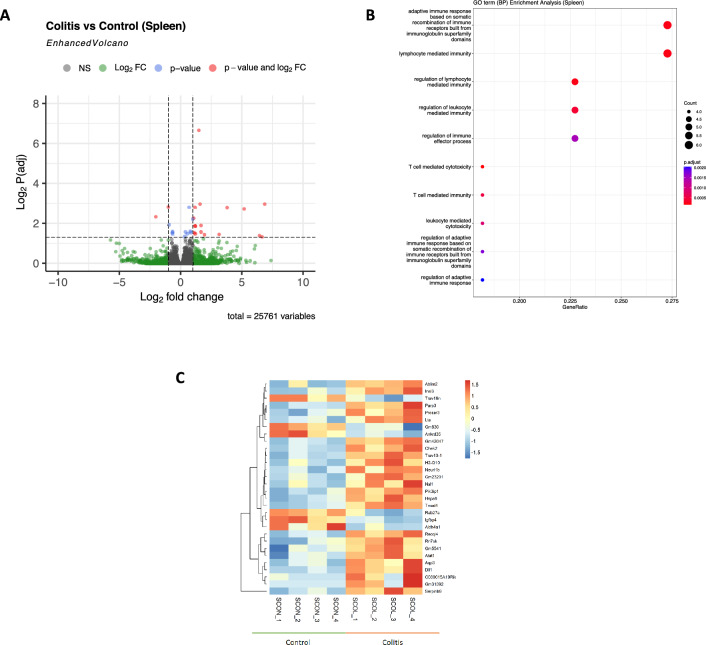
RNAseq analysis of splenic CD4^+^ T cells shows minimal variations in the context of intestinal inflammation. (**A**) Volcano plot of differentially expressed genes in CD4^+^ T cells from colitis matched to control, showing log_2_fold change vs -log_2_P (adj), using Deseq-2 algorithm. In red circles are shown genes differentially expressed. *Dashed lines* indicate the *x*-axis threshold of |log_2_[fold change]|> 2 and the *y*-axis threshold of log_2_P(adj) < 0.05. (**B**) Gene ontology enrichment analysis of gene cluster altered in splenic CD4^+^ T cells from inflamed tissue. (**C**) Heat-map of a total gene of splenic CD4^+^ T cell altered in intestinal inflammation (n = 4).

Our original aim was to discern which are the genes differentially expressed in CD4^+^ T cells from both colon and spleen, associated with a pathogenic inflammatory condition. For this purpose, we first filtered the transcripts that displayed at least a two-fold difference in the colitis group compared with the respective control group. Volcano plot of DEG from colonic lamina propria CD4^+^ T cells (Fig. 2A) revealed that most DEG were up-regulated upon inflammatory colitis. Afterward, we analyzed the biological processes associated with DEGs performing a gene ontology enrichment analysis (Fig. 2B). We found numerous pathways related with inflammation such as leukocyte migration, chemotaxis, wound healing, regulation of the inflammatory response, in addition with those related with cytokine response and cell proliferation. Importantly, at least 15 genes were affected in each process described. To summarize this data, we display a heatmap with key DEG clusterizing into Th1 response, leukocyte migration, and response to cytokines in the colonic CD4^+^ T cells collected from inflammatory colitis and control conditions (Fig. 2C). Similar to the analysis of DEG in colonic CD4^+^ T cells, we compared gene expression in splenic CD4^+^ T cells. The volcano plot of this analysis (Fig. 3A) shows just a few transcripts up-regulated. The analysis of gene ontology enrichment detected only a few pathways related with the inflammation, such as adaptive response and lymphocyte mediated immunity, however no more than six genes were linked to each process (Fig. 3B). The heat map of the whole set of DEG in spleen tissue (Fig. 3C) confirms the expression pattern shown in the volcano plot, even if, as happens with colon data, there are some inconsistencies between replicates. The overall analysis of DEG suggests that colonic CD4^+^ T cells were more disturbed in colitis with several inflammatory immune responses activated, in contrast to splenic CD4^+^ T cells.

### RNA-seq network analysis of colonic CD4^+^ T cells reveals five master gene regulators involved in colitis

**Figure 4:**
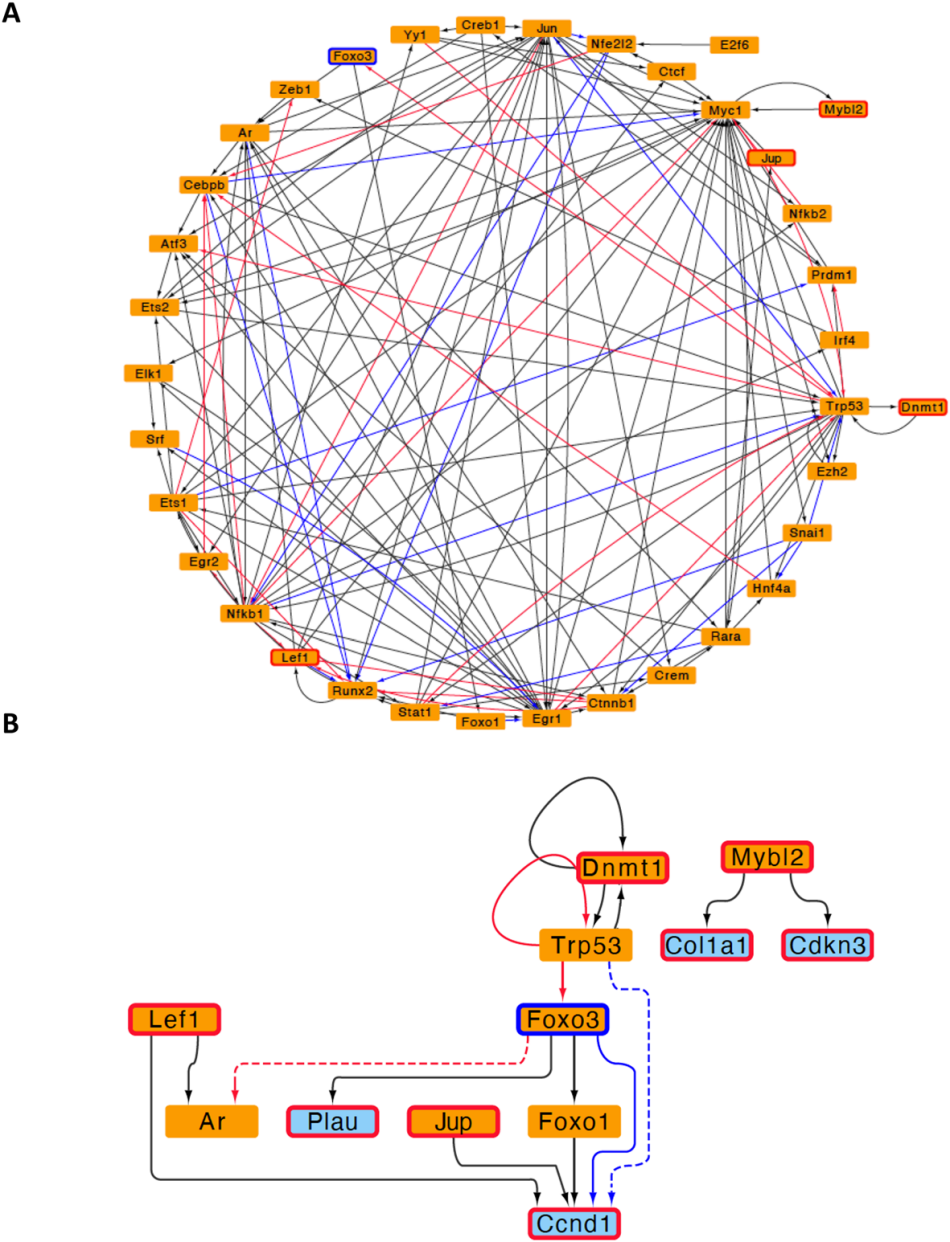
Network analysis using RNAseq data from colonic CD4^+^ T cells reveals five master regulators involved in the pathogenic profile of colitogenic effector T cells. (**A**) Regulatory network of 36 master regulators involved in the pathogenic profile of effector CD4^+^ T cells isolated from the inflamed colonic lamina propria. (**B**) A brief summary of the network of the five master regulators (*Lef1, Jup, Mybl2, Dnmt1* and *Foxo3*) altered in inflamed condition. Master regulators (MR) are represented in orange rectangles, while key gene controlled by MR are represented as blue rectangles. Red border represents up-regulation and blue border represents down-regulation of MR in colitis relative to control.

As we have shown above, an intricate gene regulatory network was observed in colonic CD4^+^ T cells under inflammatory conditions in a colitis mouse model. Indeed, a list of key master regulators have been described to drive CD4^+^ T cell differentiation into Th1 and Th17 inflammatory phenotypes^9^. Hence, we hypothesize whether a limited number of master regulators might underlie the observed inflammatory phenotype in colitis. To achieve this objective, we developed context specific networks for both, colitis and control conditions. We identified the subnetwork of master gene regulators responsive for the observed changes in gene expression associated with colitis in colonic CD4^+^ T cells. From this system’s biology approach, we observed that the set of master gene regulators derived from colitis and control conditions are the same, which is depicted in Fig. 4A. This highlights the principal master regulators associated with the altered gene expression in colitogenic CD4^+^ T cells. From the 36 master regulators identified as driving forces behind the inflammatory condition, only five exhibited differential expression in this context. Figure 4B illustrates the relationships among these five master regulator genes through the selection of their first predecessor in the network, involving *Lef1, Jup, Dnmt1,* and* Mybl2* which were up-regulated, and *Foxo3* that was down-regulated. The other master regulators displayed (*Trp53, Ar1,* and* Foxo1*) were only associated with the previously mentioned genes, but no differential expression was observed. Additionally, we conducted a similar gene network analysis using data from splenic CD4^+^ T cells. Notably, the minimal gene perturbation observed in these cells resulted in a more constrained network, as illustrated in Fig. S2. None of the seven candidates to be master regulators identified within this network exhibited significant differential expression, indicating that further data might be required to complete the analysis in splenic cells. Altogether, these analyses suggest that only the five identified master gene regulators associated with the colitis inflammatory condition could be responsible for the altered phenotype observed in colonic CD4^+^ T cells.

### *Lef1* and *Mybl2* were found to be up-regulated, highlighting them as potential key candidates in IBD research

**Figure 5:**
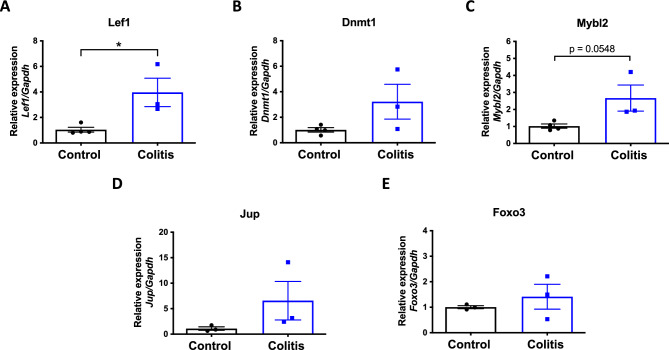
Lef1 and Mybl2 are up-regulated in colonic CD4^+^ T cells during colitis. Levels of master regulator transcripts were analysed by qRT-PCR. (**A**) *Lef1* (**B**) *Dnmt1* (**C**) *Mybl2* (**D**) *Jup* and (**E**) *Foxo3*. The levels of *Gapdh* transcripts were used as a housekeeping. Data were obtained from 3–4 mice per group. Each symbol represents data obtained from an individual mouse. N = 3–4, mean ± SEM are indicated. *, *p* < 0.05 by student’s t-test.

Above, we identified five master regulators potentially defining the pathogenic phenotype of CD4^+^ T cells situated in the colonic lamina propria upon inflammatory colitis. To verify the differential expression delineated by bulk RNAseq analysis for these transcription factors, we carried out the transcripts quantification by qRT-PCR using counterpart samples from the RNA isolated from each replicate. The outcomes of this validation are presented in Fig. 5 (see the raw data in supplementary information), where distinct expression trends of the examined genes can be distinguished. *Lef1* (Lymphoid enhancer-binding factor 1) and *Mybl2* (Myb-related protein B) exhibited a statistically significant up-regulation, exceeding threefold the control levels (Fig. 5A,C). *Dnm1* (DNA-methyltransferase 1) and *Jup* (Junction plakoglobin) showed a trend towards an increase, without reaching a statistical significance (Fig. 5B,D). Unexpectedly, *Foxo3* (Foxkhead box protein O3) did not show down-regulation; instead, a subtle upward trend was observed, suggesting its potentially limited influence within the outlined regulatory network (Fig. 5E). Altogether, these findings suggest that *Lef1* and* Mybl2* stand out as potential key regulator genes in CD4^+^ T cell biology requiring further exploration in IBD research.

## Discussion

In our study, we integrated bulk RNA-seq transcriptomic analysis with a gene regulatory network approach to thoroughly characterize conventional CD4^+^ T cells from the colonic lamina propria and the spleen using a transfer colitis mouse model. We opted for this model due to its chronic manifestation of gut inflammation over a time of ten to twelve weeks^33^, as opposed to mouse models of acute inflammation induced by the administration of dextran sulfate sodium^32^. Additionally, the proliferation of lymphocytes in the recipient mouse facilitates tracking specific cell markers^33,34^. Our findings revealed that CD4^+^ T cells from the colonic lamina propria upon gut inflammation, display a proinflammatory profile, with notable alterations in leukocyte migration, cytokine response, and Th1 response pathways. In contrast, splenic CD4^+^ T cells presented a less perturbed profile upon intestinal inflammation, with only a limited number of genes affected. Given these findings, our attention was directed towards the changes in the colon to model the master regulator network behind the inflammatory state. We identified a set of five transcription factors (*Lef1, Jup, Mybl2, Dnmt1*, and* Foxo3*) with differential expression that seem to orchestrate the pathogenic profile of colonic CD4^+^ T cells involved in gut inflammation. Furthermore, we confirmed the upregulation of* Lef1* and *Mybl2* in CD4^+^ T cells, suggesting that these master regulators might play a pivotal role in the progression of the disease. Of note, the master regulators found in CD4^+^ T cells in this study are not necessarily involved exclusively in gut inflammation. Some of these master regulators might be related to many other processes, especially those that were not differentially expressed, including *Trp53* and *Ar1*, which are known to be cell cycle master regulators^35,36^. Another limitation is that this study was conducted only with females since male mice behave highly aggressive when housed in groups^37^. Importantly, single housing is not a good option to avoid intermale aggressions, as single housing might induce significant behavioral and physiological changes through the hypothalamus–pituitary–adrenal axis dysregulation^38,39^, ultimately affecting intestinal immunity^40^.

In the transfer models of colitis, both Th1 and Th17 cells have been associated with the pathogenic gut inflammation^41^. Treg also infiltrate the colonic lamina propria and participate in inflammatory colitis, limiting inflammation^42^. In our experimental approach, we selectively isolated effector CD4^+^ T cells, excluding Treg from the analysis. We focused on effector CD4^+^ T cells due to their central role in the development of intestinal inflammation in the colitis transfer model. The transcriptomic data we obtained represents an average of the subsets present during sample collection, and the signals we detected could potentially be masked by background noise. Although our analysis was focused on Teff, the significance of Treg in the development of inflammatory colitis cannot be ignored. An interesting example of this is a recent study using a colitis model induced by transferring naïve CD4^+^ T cells into *Rag2*^-/-^ mice. These authors found that the transfer of naïve T cells deficient in the Zinc finger protein (Zfp)362 displayed a less severe colitis, which was associated with a reduction of Treg function rather than a higher promotion of Th17 differentiation^43^.

Of note, previous studies have explored several immune populations in colonic mucosal samples from IBD patients, including UC and CD using single-cell RNAseq. Mitsialis and colleagues highlighted that unique immune cell signatures that characterize each of these particular disorders. Also, they observed an increase of memory Treg cells in the intestinal mucosa of IBD patients, which also exhibited proinflammatory cytokine expression^44^. Moreover, naïve T cells, Treg, Th17 and highly activated T cells have been found increased in the inflamed intestinal mucosa from CD patients^45,46^. A similar study conducted on the intestinal lamina propria of CD patients revealed an increase in CD8^+^ T cells, along with a decrease in CD4^+^ T cells. This change was also paralleled by an elevated Th17-to-Treg ratio^47^. Recently, a complete map of cellular composition in colonic CD tissue was published, showing overrepresentation of CD4^+^ and CD8^+^ T cells in the intraepithelial compartment, a distribution that evolves during the inflammation process^48^. Additionally, the expression profile of peripheral blood Treg was evaluated in IBD patients undergoing vedolizumab treatment, a monoclonal antibody that blocks α4β7 integrin, thus dampening the recruitment of lymphocyte into the intestine. Interestingly, the therapeutic antibody treatment demonstrated a significant impact on Treg metabolic pathways, particularly oxidative phosphorylation, which is associated with better bioenergetic fitness and higher Treg suppressive function and persistence^49^.

Regarding our approach for the analysis of gene regulatory networks, we identified five master regulators involved in colonic inflammation, being *Lef1* and *Mybl2* the main candidates. Lef1 is a member of the Tcf/Lef transcription factor family and integral to Wnt signaling cascades^50^. Indeed, Lef1 is indispensable during thymic T cell maturation, especially in the double-negative to double-positive transition^51^. Lef1 is required for IL-17A-expressing γδT-cell maturation and development, and is essential in the immunosuppressive function of regulatory T cells^52,53^. Furthermore, Lef1 has been shown to predominantly be expressed by Th1 cells, and to interact with the transcription factor Gata-3, regulating Gata-3 function (e.i. reduction of Th2 cytokines IL-4, IL-5, and IL-13)^54,55^. Thus, suggesting that Lef1 acts as a repressor for gene expression in T cells. A recent study has shown that a novel population of T-bet experience naïve-like CD4^+^ T cells have increased Lef1 expression compared to effector memory T cells. This T cell population produces high levels of IFN-γ upon activation and exhibit a Th1 polarization phenotype. The authors suggest that this subset of CD4^+^ T cells are able to produce a less severe colitogenic phenotype due to the lack of IL-17-A production^56^. Mybl2, commonly termed B-Myb, is a pivotal transcription factor within the Myb protein family, involved in cell cycle modulation, predominantly during the S phase and G2/M transition^57,58^, cell differentiation and proliferation^59^. Mybl2 have been related with infiltration of immune cells in several types of cancer. For instance, it correlates with immune infiltrates in prostate cancer^60,61^. Furthermore, increased Mybl2 expression has been correlated with poor patients survival in different types of cancer^59^. Concerning to the other upregulated genes in colonic CD4^+^ T cells upon intestinal inflammation, *Dnmt1* and *Jup*, just a few evidences have been associated to immune activation. Dnmt1 contributes to DNA methylation and is required for the proper expression of certain genes that determine T cell fate and function^62^. Jup upregulation have been reported in inflamed bowel mucosa^63^.

Remarkably, Lef1 expression has been observed increased in single-cell RNAseq analyses of colonic CD4^+^ lymphocytes from UC patients^64^. Furthermore, a single-cell landscape study of adaptive lymphocytes in pediatric IBD from the least inflamed areas of the colon identified Lef1 expression in both CD8^+^ and naïve CD4^+^ T cells^65^. Niclosamide, an FDA-approved anti-helmintic drug, downregulates Wnt signaling pathway by suppressing Lef1 expression, which plays a pivotal role in regulating cellular stemness^66^. Intriguingly, niclosamide has been shown to effectively reduce acetic acid-induced colitis in rats^67^. Additionally, a phase I clinical trial involving a niclosamide enema is currently underway for ulcerative colitis patients^68^.

## Conclusions

Our findings suggest that the pathogenic profile of effector CD4^+^ T cells in the colonic lamina propria under inflammatory conditions is controlled by a few overexpressed master regulators. Specifically, we propose that *Lef1* and *Mybl2* play a crucial role in the pathogenic commitment of CD4^+^ T cells. These findings encourage further research of these master regulator genes in the context of IBD pathogenesis and to consider them as potential molecular targets for therapeutic interventions in IBD.

### Supplementary Information

Supplementary Information 1.

Supplementary Figures.

Supplementary Information 2.

Supplementary Information 3.

Supplementary Table S1.

Supplementary Table S2.

## Data Availability

The datasets generated and analyzed during the current study are available in the National Center for Biotechnology Information (NCBI) repository, using the identifier PRJNA1063770.
