# Supplementary material for: Identification of master regulator genes controlling pathogenic CD4+ T cell fate in inflammatory bowel disease through transcriptional network analysis
Source: Sci Rep. 2024 May 8;14:10553. doi: 10.1038/s41598-024-61158-4 (PMC11078927; doi:10.1038/s41598-024-61158-4)
Supplement: Supplementary file 2 — Supplementary Figures. [file 41598_2024_61158_MOESM2_ESM.pdf]

Figure S1

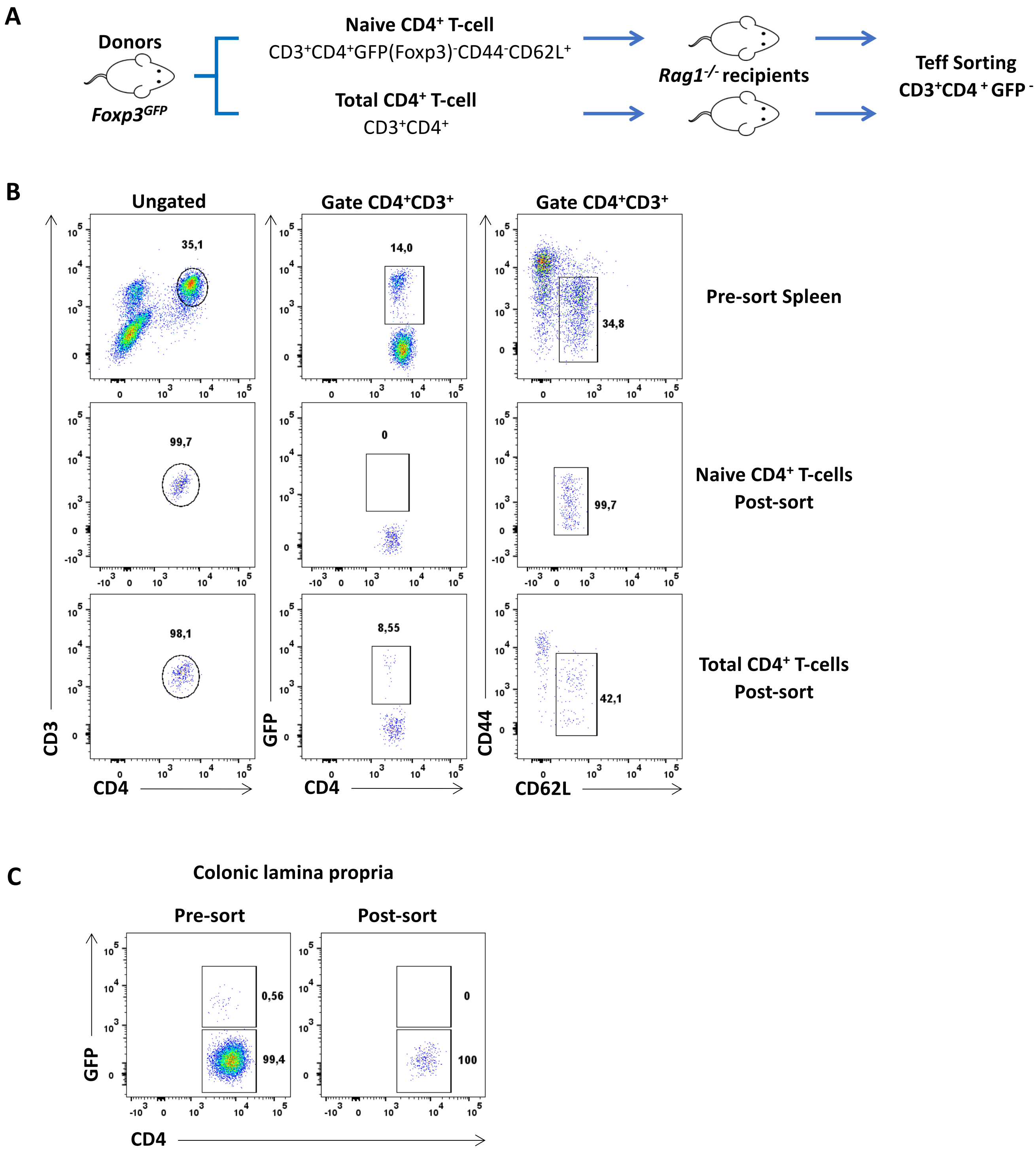

Figure Supplementary 1. Flow cytometry analysis of CD4$^+$ T cells before and after cell-sorting. *Rag1*$^{-/-}$ recipient mice received the i.v. transfer of naïve CD4$^+$ T cells or total CD4$^+$ T cells (5 x10^5 cells per mouse) as control. After 10 to 12 weeks, mice were euthanized to obtain lymphocytes from spleen and colonic lamina propria, and Teff were purified by cell-sorting. (**A**) Scheme illustrating the experimental strategy. (**B**) Representative dot-plots before and after cell sorting of donor T cells previous to the i.v. injection into recipient mice. (**C**) Representative dot-plots before and after cell sorting of Teff obtained from the colonic lamina propria. (B and C) The percentage of cells inside the regions are indicated.

Figure S2

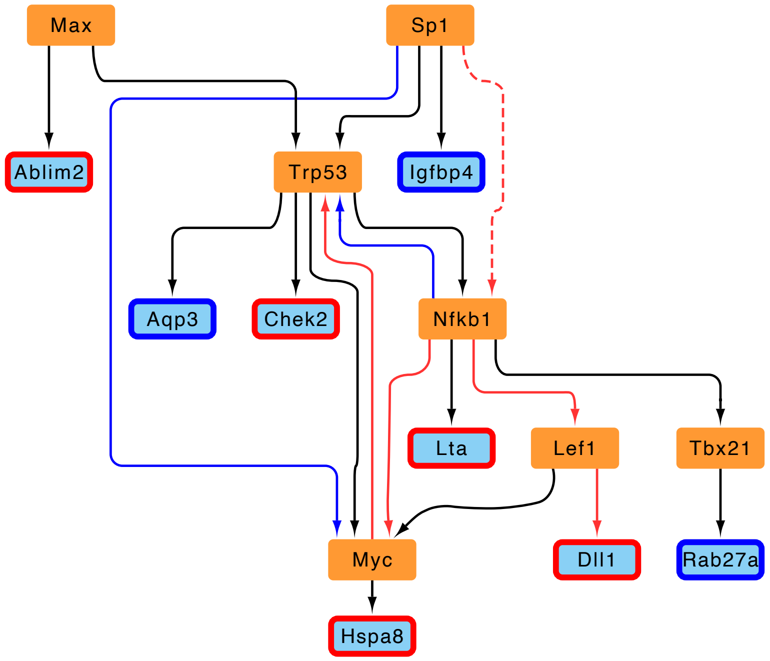

Figure Supplementary 2. Network analysis using RNAseq of splenic CD4⁺ T cells. Regulatory network of seven candidates to master regulators implicated in the gene expression of splenic CD4⁺ T cells in colitis. Master regulators (MR) are represented in orange rectangles, while key gene controlled by MR are represented as blue rectangles. Red border represents up-regulation and blue border represents down-regulation of MR in colitis relative to control.
